# Associations between the Dietary Inflammatory Index and depression among pregnant and postpartum women: analysis of NHANES 2005–2018

**DOI:** 10.3389/fnut.2025.1681491

**Published:** 2025-11-27

**Authors:** Jian Qiao, Aiqing Han, Yannv Qu, Li Zhang

**Affiliations:** 1 Psychiatry Department, Jinan Mental Health Center, Jinan, China; 2 Department of Obstetrics and Gynecology, Jinan Maternity and Child Care Hospital, Jinan, China; 3 Geriatrics Department, Peking University Shenzhen Hospital, Shenzhen Peking University-The Hong Kong University of Science and Technology Medical Center, Shenzhen, China; 4 Shenzhen University, Shenzhen, China

**Keywords:** Dietary Inflammatory Index (DII), depression, PHQ-9, pregnant women, postpartum women, perinatal

## Abstract

**Background:**

Perinatal depression, which occurs during pregnancy or after childbirth, is a common and serious complication of pregnancy. Chronic low-grade inflammation has been implicated in the pathophysiology of depression. The Dietary Inflammatory Index (DII) is a validated measure of the inflammatory potential of a diet, where higher scores indicate a more proinflammatory diet. This study examined the association between DII and depression among pregnant and postpartum women, with a focus on potential non-linear (threshold) effects.

**Methods:**

We analyzed data from the National Health and Nutrition Examination Survey (NHANES) 2005–2018. The sample included 1,093 women who were either pregnant or in the postpartum period within 18 months. Dietary intake was assessed via 24-h recall, and DII scores were calculated for each participant. Depressive symptoms were measured using the Patient Health Questionnaire-9 (PHQ-9); we defined depression as a PHQ-9 score ≥10, indicating moderate or greater depressive symptoms. Weighted multivariate logistic regression was used to estimate odds ratios (ORs) for depression in relation to DII scores, and weighted linear regression was used to assess the association between DII scores and PHQ-9 total scores. Models were adjusted for potential confounders, including age, race, poverty income ratio, marital status, smoking status, education level, BMI and WBC. We tested for non-linear relationships using a generalized additive model (GAM). A two-piecewise linear regression model was then applied, and a log-likelihood ratio test was used to compare the piecewise model to a single-linear-term model. Sensitivity analyses were conducted to check the robustness of the findings.

**Results:**

The mean age of pregnant and postpartum women was 28.88 (95% CI 28.34–29.42) years. Overall, 7.1% participants met the criteria for moderate/severe depression (PHQ-9 score ≥10). DII scores ranged from anti-inflammatory to proinflammatory; approximately 21.9% of the sample had a highly proinflammatory diet (DII > 2.87). We observed a non-linear association between DII and depression (*P* for non-linearity = 0.008 for the threshold effect). In adjusted weighted logistic models, among women with DII > 2.87, each 1-unit increase in DII was associated with an OR = 2.81 for depression (95% confidence interval 1.07–7.38, *p* = 0.039). Consistently, in the weighted linear regression, when the DII exceeded 2.87, each additional DII point corresponded to an increase of *β* = 1.59 points in the PHQ-9 score (95% CI 0.02–3.17, *p* = 0.047), indicating worse depressive symptom severity with more proinflammatory diets. The associations remained significant and of similar magnitude in the sensitivity analyses.

**Conclusion:**

In this cross-sectional study of pregnant and postpartum women in the U. S., a higher Dietary Inflammatory Index was associated with a greater risk of depression, but this relationship was markedly non-linear. Diets with very high proinflammatory potential were linked to significantly increased odds of perinatal depression, whereas more anti-inflammatory diets did not result in a further decrease in depression risk below that threshold. However, owing to the inherent limitations of the study design, causality cannot be established, and a reverse effect of depressive symptoms on dietary choice cannot be ruled out.

## 1 Introduction

Perinatal depression is a prevalent and consequential maternal health issue. Epidemiological data indicate that as many as 1 in 7 women in the United States report significant depressive symptoms after giving birth (1), with global estimates suggesting a mean prevalence of 26.3% (2). These mood disturbances can impair maternal functioning and have long-term adverse effects on both mothers and children (3). Untreated perinatal depression is associated with poorer maternal–infant bonding, lower breastfeeding rates, and developmental delays in offspring (4, 5). In severe cases, maternal depression can even contribute to pregnancy-related morbidity and mortality (e.g., through suicide or substance abuse) (6). Identifying risk factors for perinatal depression is therefore a public health priority, as it could inform prevention and early intervention efforts.

Among the array of potential risk factors, chronic inflammation has gained attention because of its role in depression (7, 8). There is substantial evidence that diets high in refined carbohydrates, saturated fats, and processed foods tend to promote inflammation, whereas diets rich in vegetables, fruits, whole grains, and omega-3 fatty acids tend to be anti-inflammatory (9–11). This emerging field connecting nutrition, inflammation, and mental well-being suggests that diet is a modifiable factor deserving closer examination in relation to perinatal depression. To quantify the inflammatory potential of an individual’s diet, researchers have developed the Dietary Inflammatory Index (DII). A lower (more negative) DII score indicates a more anti-inflammatory diet, whereas a higher (positive) DII score reflects a proinflammatory diet. Several cross-sectional studies have reported that only women appear to be at an increased risk of recurrent depression as DII scores increase (12, 13). Yuan et al. (14) reported that compared with pregnant women in the lowest tertile, pregnant women in the highest tertile of energy-adjusted DII had significantly greater odds of prenatal depression. Zhang et al. (15) reported that a high DII is a risk factor for mid-pregnancy depression. Similarly, one U. S. study of non-pregnant adults reported a J-shaped relationship, where depression risk was especially elevated once the DII exceeded ~2.7 (16). Overall, these studies support the idea that a highly proinflammatory diet might contribute to depression, and they also indicate that the relationship is non-linear, with a potential threshold beyond which risk increases more dramatically.

Despite this growing literature, data specifically focused on dietary inflammation and depression in pregnant and postpartum women remain limited (10, 11, 14). It is important to verify whether the DII–depression association observed in other groups holds true for pregnant/postpartum women and, if so, to characterize its dose–response pattern. Such information could inform nutritional guidance as part of mental health care.

Study Objective: The present study aims to investigate the association between the Dietary Inflammatory Index (DII) and depression among pregnant and postpartum women in the United States using nationally representative NHANES data from 2005 to 2018. We hypothesized that a more proinflammatory diet (higher DII) would be associated with a greater likelihood of depressive symptoms. We also specifically tested for non-linearity in this association, given prior hints of a threshold effect, to determine whether there is a point at which the risk of depression increases more sharply with increasing DII.

## 2 Methods

### 2.1 Study population and data source

This study utilized data from the National Health and Nutrition Examination Survey (NHANES), years 2005–2018. The NHANES is a continuous cross-sectional survey conducted by the U. S. National Center for Health Statistics that is designed to assess the health and nutritional status of the civilian, non-institutionalized population. It employs a complex multistage probability sampling design to generate a nationally representative sample. Participants undergo interviews, physical examinations, and laboratory tests. In the present analysis, we combined seven two-year NHANES cycles (2005–06, 2007–08, …, 2017–18) to obtain a sufficient sample of pregnant and postpartum women.

**Figure 1:**
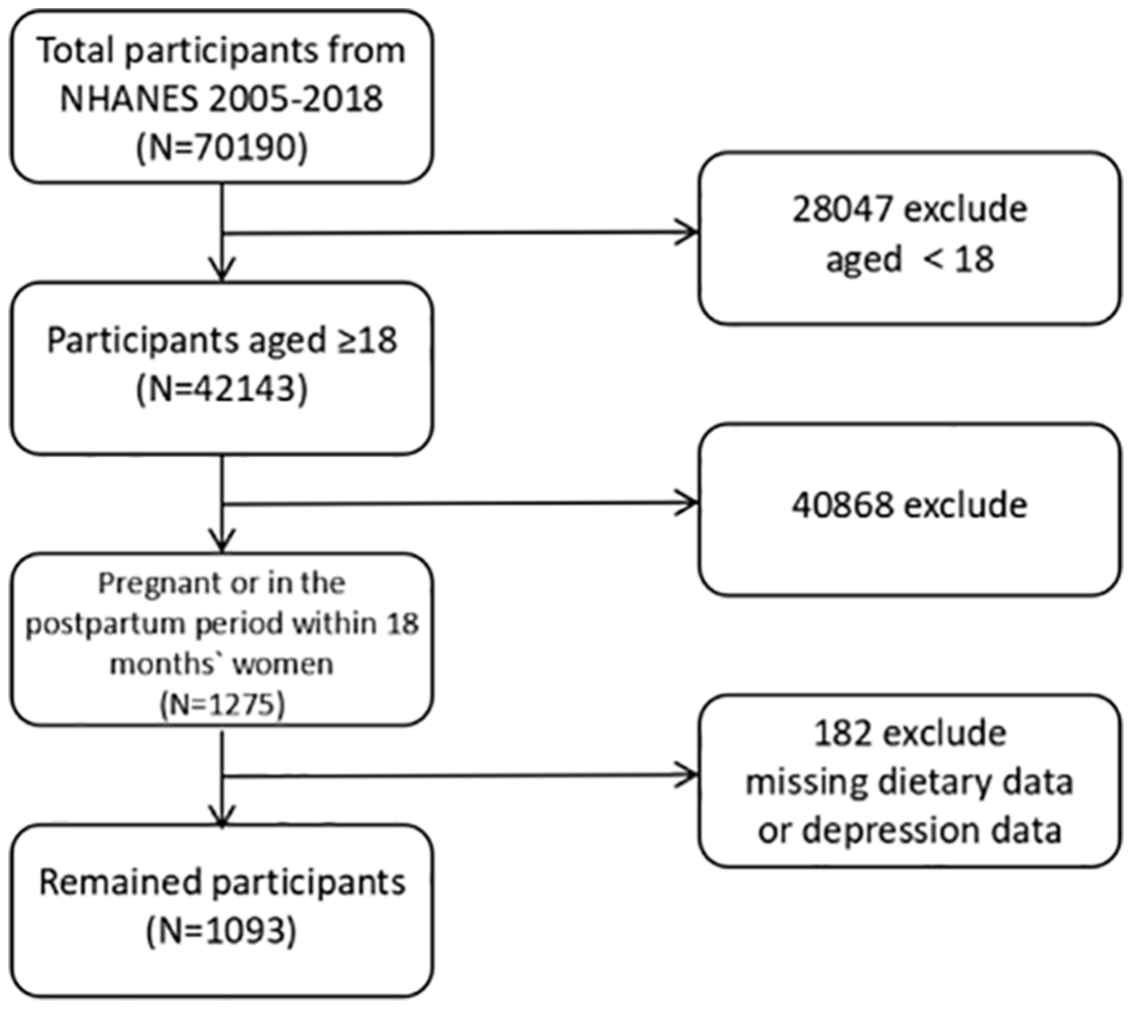
Flow chart of participant selection from the NHANES 2005–2018.

The inclusion criteria for our study were female participants of reproductive age who were either pregnant or in the postpartum period within 18 months. Pregnancy status in NHANES was determined by self-report and a pregnancy test as part of the examination. We considered a woman postpartum based on her report of having given birth within the past 18 months, as captured by the interview data on reproductive history. Women who were neither currently pregnant nor recently postpartum were excluded from this analysis. We also excluded any individuals with missing data on dietary intake or depression symptoms. After applying these criteria, our final sample consisted of *N* = 1,093 women (Figure 1).

### 2.2 Dietary Inflammatory Index (exposure)

Dietary intake was assessed in the NHANES via a 24-h dietary recall interview administered by trained personnel. Participants detailed all foods and beverages consumed in the preceding 24 h (midnight to midnight). Nutrient intakes were then estimated using the USDA Food and Nutrient Database. For each participant, we computed the Dietary Inflammatory Index (DII) to quantify the inflammatory potential of their diet.

The DII was calculated according to the method developed by Shivappa et al. (17). For each participant, we calculated the DII from 28 food parameters. Carbohydrates, protein, total fat, saturated fat, alcohol, fiber, cholesterol, MUFAs, PUFAs, *n*-3 fatty acids, *n*-6 fatty acids, niacin, vitamin A, thiamin (vitamin B1), riboflavin (vitamin B2), vitamin B6, vitamin B12, vitamin C, vitamin D, vitamin E, Fe, Mg, zinc, selenium, folic acid, beta-carotene, caffeine, and energy were used to quantify the inflammatory potential of their diet (Supplementary Table 1 shows the data for the calculation of DII). To calculate the DII scores, first, *Z* score = (daily intake of a certain dietary ingredient or nutrient–global daily mean intake)/the standard deviation of the global mean per capita daily intake of this dietary ingredient or nutrient. Then, the *Z* score was converted to a percentile scale, the obtained percentile value was doubled, and “1” was subtracted to achieve a symmetrical distribution centered on “0.” Finally, the total inflammation score of each dietary component was multiplied to calculate the inflammation index of each dietary component or nutrient, thereby yielding the individual’s DII (17–19).

A positive DII score indicates a diet relatively high in proinflammatory components, whereas a negative DII score indicates a diet rich in anti-inflammatory components. For example, high consumption of saturated fat or added sugars would increase the DII, whereas high consumption of fiber, magnesium, or omega-3 fatty acids would lower the DII, based on evidence of their relationships with inflammatory markers. In our dataset, DII was treated as a continuous variable for analysis. We also examined the distribution of DII and noted that approximately the top quintile of the sample had notably high DII values (approximately +3 or greater), which helped inform our investigation of a potential threshold effect.

### 2.3 Depression assessment (outcome)

The outcome of interest was depression status or depressive symptom severity, measured by the Patient Health Questionnaire-9 (PHQ-9) instrument. The PHQ-9 questionnaire was developed to assess each of the nine diagnostic criteria for major depression as outlined by the Diagnostic and Statistical Manual of Mental Disorders, fourth edition, in terms of the effect of each symptom on the participant in the 2 weeks prior to the survey (20). Each item is scored from 0 (“Not at all”) to 3 (“Nearly every day”), and the PHQ-9 total score ranges from 0 to 27. In the NHANES, the PHQ-9 was administered as part of the mental health questionnaire for participants 18 and older (and for adolescents 12–17 with a youth version). For our study, we utilized the adult PHQ-9 results for the women in our sample. We defined depression as a PHQ-9 score ≥10, which corresponds to at least moderate depression in the established severity categories. This cutoff (PHQ-9 ≥ 10) is widely used to indicate clinically significant depressive symptoms and has good sensitivity and specificity for major depressive disorder in validation studies. We chose this binary definition to identify women with clinically relevant depression symptoms. In addition, we treated the PHQ-9 total score as a continuous outcome in secondary analyses to assess the association between the DII score and overall depression symptom severity. Using both binary and continuous outcomes allowed us to capture the presence of depressive disorder as well as more subtle changes in symptom levels.

### 2.4 Covariates

We adjusted for a range of covariates that might confound the relationship between diet and depression based on prior literature and data availability in the NHANES. The following variables were included in the multivariable models:

Age (in years, continuous) – maternal age at the time of the survey.

Race/ethnicity (categorical) – self-reported race/ethnicity, categorized as Non-Hispanic White, Non-Hispanic Black, Hispanic (including Mexican American and other Hispanic), and Other (including Asian and multiracial), as defined by NHANES. This accounts for potential ethnic differences in diet or depression prevalence.

Marital status (categorical) – categorized as married/cohabiting, never married, or separated/divorced/widowed, as marital or partner support may be related to both diet and mental health.

Poverty-income ratio (PIR) (continuous) is the ratio of household income to the federal poverty level, which is an index of socioeconomic status (SES). Lower PIR (indicating lower income relative to needs) has been associated with both poorer diet quality and higher depression risk; thus, adjusting for SES is important.

Education level (categorical) – highest attained education (below high school, high school, or above high school). Education is another SES indicator and may influence health literacy, dietary choices, and mental health.

Smoking status (categorical) – classified as current smoker, former smoker, or never smoker. Smoking can induce inflammation and is also associated with depression; thus, smoking was included to reduce confounding.

Body mass index (BMI) (continuous) – determined by dividing a person’s weight (in kilograms) by the square of their height (in meters).

WBC (1,000 cells/μL, continuous) – White blood cell count from laboratory data of the NHANES.

## 3 Statistical analysis

We first performed descriptive analyses to characterize the study sample. To enhance the precision of the data and thus minimize the impact of the intricate multi-stage sampling design employed in NHANES, the research applied sample weights as recommended by the NHANES guidelines. The continuous variables are represented as survey-weighted mean (95% CI), while the categorical variables are represented as survey-weighted percentage (95% CI) (21).

Then, we performed weighted univariate logistic and weighted univariate linear regression analyses, and depression and PHQ-9 total scores were examined separately according to age, race, poverty income ratio, marital status, smoking status, education level, DII, BMI, WBC count and other variables.

For our primary analysis, we used weighted multivariable logistic regression to examine the association between the DII and the odds of depression (binary outcome). In these models, the outcome variable was depression = 1 if the PHQ-9 score was ≥10 and 0 otherwise. The main independent variable was DII (per one-unit increase). We fitted an initial model treating DII as a continuous linear variable and including all the covariates listed above. This provided an overall OR for depression per unit increase in DII, adjusted for confounders. However, given prior evidence suggesting a non-linear relationship (16), we did not assume that linearity was the true form of the association.

Based on the prior literature, we hypothesized a threshold at DII ≈ 2.8–2.9 beyond which the association becomes stronger (16). We applied a two-piecewise linear model, also known as segmented regression or change-point analysis, using the methods described by Zhao et al. (16). A likelihood ratio test was conducted to compare this two-piecewise model to a simpler model with only a single linear term for DII.

For the secondary analysis, we ran weighted linear regression models with the PHQ-9 total score as the outcome (a continuous measure of depressive symptom severity). We mirrored the approach above: first, linear vs. non-linear fit was assessed, and then a piecewise linear model with the same threshold (DII 2.87) was used for consistency. The beta coefficients (*β*) from these models indicate the difference in the PHQ-9 score associated with each one-unit difference in DII in the low and high DII ranges.

All regression models were adjusted for the full set of covariates, including age, race, poverty income ratio, marital status, smoking status, education level, BMI, and WBC. We conducted several sensitivity analyses to ensure the robustness of our findings. Statistical significance was set at *α* = 0.05 (two-tailed). All analyses were performed using R (version 4.2.0) and EmpowerStats^1^.

## 4 Results

### 4.1 Participant characteristics

A total of 1,093 women (pregnant or within 18 months post-partum) from NHANES 2005–2018 were included. Table 1 shows the demographic and clinical characteristics of the participants according to the baseline DII in three tertiles: low (−3.49–0.72), middle (0.72–2.34), and high (2.34–4.63). The mean age was 28.88 years (95% CI 28.34–29.42, range ~18–47). The mean of the PHQ-9 total score was 3.24 (95% CI 2.93–3.55), and approximately 7.10% perinatal women have a PHQ-9 score ≥10.

**Table 1:** Demographic and clinical characteristics of the participants, weighted.

	Total	DII			*P* value
		Low (−3.49–0.72)	Middle (0.72–2.34)	High (2.34–4.63)	
Age (years)	28.88 (28.34,29.42)	30.30 (29.48,31.12)	28.04 (27.23,28.85)	28.25 (27.28,29.21)	0.0003
BMI (kg/m^2^)	29.60 (28.96,30.24)	28.60 (27.52,29.68)	29.93 (28.84,31.02)	30.32 (29.14,31.50)	0.1154
DII	1.43 (1.27,1.59)	−0.54 (−0.65,-0.43)	1.56 (1.51,1.62)	3.29 (3.21,3.36)	<0.0001
WBC (1,000 cells/μL)	8.25 (8.07,8.43)	7.95 (7.59,8.32)	8.60 (8.25,8.95)	8.21 (7.86,8.57)	0.0583
PHQ-9 total score	3.24 (2.93,3.55)	2.87 (2.40,3.35)	3.00 (2.54,3.46)	3.84 (3.25,4.44)	0.0312
Race/ethnicity (%)					0.0072
Non-Hispanic White	57.04 (51.35,62.54)	58.77 (51.48,65.70)	54.92 (47.07,62.53)	57.28 (49.14,65.05)	
Non-Hispanic Black	12.70 (10.03,15.94)	9.54 (6.57,13.65)	11.32 (8.20,15.43)	17.18 (12.32,23.46)	
Mexican American	14.09 (11.53,17.11)	14.51 (10.70,19.39)	16.06 (11.95,21.25)	11.81 (8.88,15.53)	
Other Hispanic	7.16 (5.51,9.26)	5.58 (3.54,8.70)	7.68 (5.49,10.64)	8.28 (5.31,12.70)	
Other Race	9.01 (7.22,11.19)	11.60 (8.21,16.13)	10.02 (6.81,14.49)	5.45 (3.82,7.71)	
Marital status (%)					0.2037
Married/Living with partner	80.83 (77.46,83.80)	84.63 (79.20,88.85)	80.94 (74.92,85.79)	76.87 (70.83,81.98)	
Widowed/Divorced/Separated	3.97 (2.87,5.46)	3.75 (1.97,7.04)	3.40 (1.95,5.86)	4.73 (2.86,7.72)	
Never married	15.20 (12.71,18.09)	11.61 (8.16,16.27)	15.66 (11.46,21.04)	18.40 (13.92,23.92)	
Poverty income ratio (%)					0.0008
Poor	23.49 (20.69,26.54)	18.14 (13.89,23.33)	19.41 (15.10,24.58)	32.76 (27.11,38.96)	
Nearly poor	21.07 (18.00,24.51)	18.39 (14.17,23.53)	22.64 (17.38,28.94)	22.29 (17.63,27.76)	
Middle income	25.73 (22.24,29.57)	28.59 (22.61,35.43)	28.05 (22.01,34.99)	20.65 (15.44,27.05)	
High income	22.93 (19.18,27.17)	29.21 (23.23,36.00)	20.47 (14.89,27.48)	18.92 (13.31,26.18)	
Missing	6.78 (5.22,8.76)	5.67 (3.45,9.19)	9.43 (6.16,14.17)	5.38 (3.06,9.31)	
Education level (%)					<0.0001
Below high school	3.54 (2.56,4.87)	2.57 (1.27,5.11)	2.93 (1.75,4.87)	5.09 (3.22,7.96)	
High school	33.36 (29.77,37.14)	19.94 (15.71,24.98)	37.51 (30.92,44.60)	42.99 (36.24,50.01)	
Above high school	63.11 (59.21,66.83)	77.49 (72.07,82.12)	59.56 (52.37,66.36)	51.92 (44.91,58.85)	
Smoking status (%)					<0.0001
Never	70.10 (65.81,74.06)	80.29 (74.73,84.87)	69.18 (62.08,75.48)	60.67 (53.63,67.29)	
Former	13.66 (11.04,16.79)	12.44 (8.59,17.69)	16.73 (11.94,22.96)	12.00 (8.59,16.51)	
Current	15.65 (12.72,19.10)	7.03 (4.60,10.62)	13.68 (9.36,19.56)	26.22 (20.76,32.53)	
Missing	0.59 (0.28,1.24)	0.24 (0.10,0.55)	0.41 (0.14,1.17)	1.12 (0.35,3.49)	
Depression (%)					0.0331
No	92.90 (90.68,94.62)	94.46 (90.33,96.89)	95.08 (90.89,97.40)	89.26 (84.87,92.49)	
Yes	7.10 (5.38,9.32)	5.54 (3.11,9.67)	4.92 (2.60,9.11)	10.74 (7.51,15.13)	

BMI, body mass index; DII, Dietary Inflammatory Index; WBC, white blood cell.

For continuous variables: survey-weighted mean (95% CI), *P*-value was by survey-weighted linear regression. For categorical variables: survey-weighted percentage (95% CI), *P*-value was by survey-weighted Chi-square test.

### 4.2 Association of DII with depression (linear vs. non-linear analyses)

Univariate analysis indicated that age, marital status, smoking status, poverty income ratio, BMI, and DII were all significantly associated with the risk of perinatal depression (all *p* < 0.05) (Table 2).

**Table 2:** Associations between clinical characteristics and depression, weighted.

	PHQ-9 total score		Depression	
	β (95% CI)	*P*-value	OR (95% CI)	*P*-value
Age (years)	−0.07 (−0.12, −0.02)	0.0113	0.94 (0.89, 1.00)	0.0489
Race/ethnicity				
Non-Hispanic White	Ref.		Ref.	
Non-Hispanic Black	0.33 (−0.41, 1.07)	0.3853	1.23 (0.59, 2.56)	0.5904
Mexican American	0.17 (−0.52, 0.87)	0.6304	1.14 (0.59, 2.22)	0.6943
Other Hispanic	−0.33 (−0.96, 0.31)	0.3157	0.42 (0.16, 1.06)	0.0701
Other Race	−0.98 (−1.63, −0.33)	0.0040	0.55 (0.19, 1.55)	0.2604
Marital status				
Married/Living with partner	Ref.		Ref.	
Widowed/Divorced/Separated	1.44 (−0.67, 3.55)	0.1842	4.43 (1.52, 12.92)	0.0076
Never married	1.58 (0.66, 2.49)	0.0010	3.94 (2.00, 7.77)	0.0001
Poverty income ratio				
Poor	Ref.		Ref.	
Nearly poor	−0.11 (−0.95, 0.74)	0.8041	0.93 (0.49, 1.77)	0.8248
Middle income	−0.71 (−1.50, 0.07)	0.0769	0.53 (0.24, 1.18)	0.1211
High income	−1.26 (−1.91, −0.61)	0.0002	0.14 (0.03, 0.60)	0.0092
Missing	0.86 (−1.34, 3.05)	0.4461	1.31 (0.45, 3.78)	0.6170
Education level				
Below high school	Ref.		Ref.	
High school	−0.21 (−1.91, 1.50)	0.8105	0.54 (0.19, 1.55)	0.2534
Above high school	−1.15 (−2.82, 0.53)	0.1822	0.36 (0.13, 0.97)	0.0470
Smoking status				
Never	Ref.		Ref.	
Former	0.72 (−0.07, 1.51)	0.0773	2.03 (0.86, 4.81)	0.1090
Current	2.48 (1.40, 3.56)	<0.0001	3.93 (1.83, 8.42)	0.0007
Missing	0.73 (−0.44, 1.89)	0.2247	0.68 (0.05, 9.53)	0.7786
BMI (kg/m^2^)	0.08 (0.03, 0.13)	0.0022	1.04 (1.00, 1.07)	0.0441
DII	0.26 (0.07, 0.44)	0.0080	1.26 (1.02, 1.55)	0.0369
WBC (1,000 cells/μL)	0.10 (−0.02, 0.22)	0.0987	1.03 (0.93, 1.12)	0.5958

BMI, body mass index; DII, Dietary Inflammatory Index; WBC, white blood cell.

**Figure 2:**
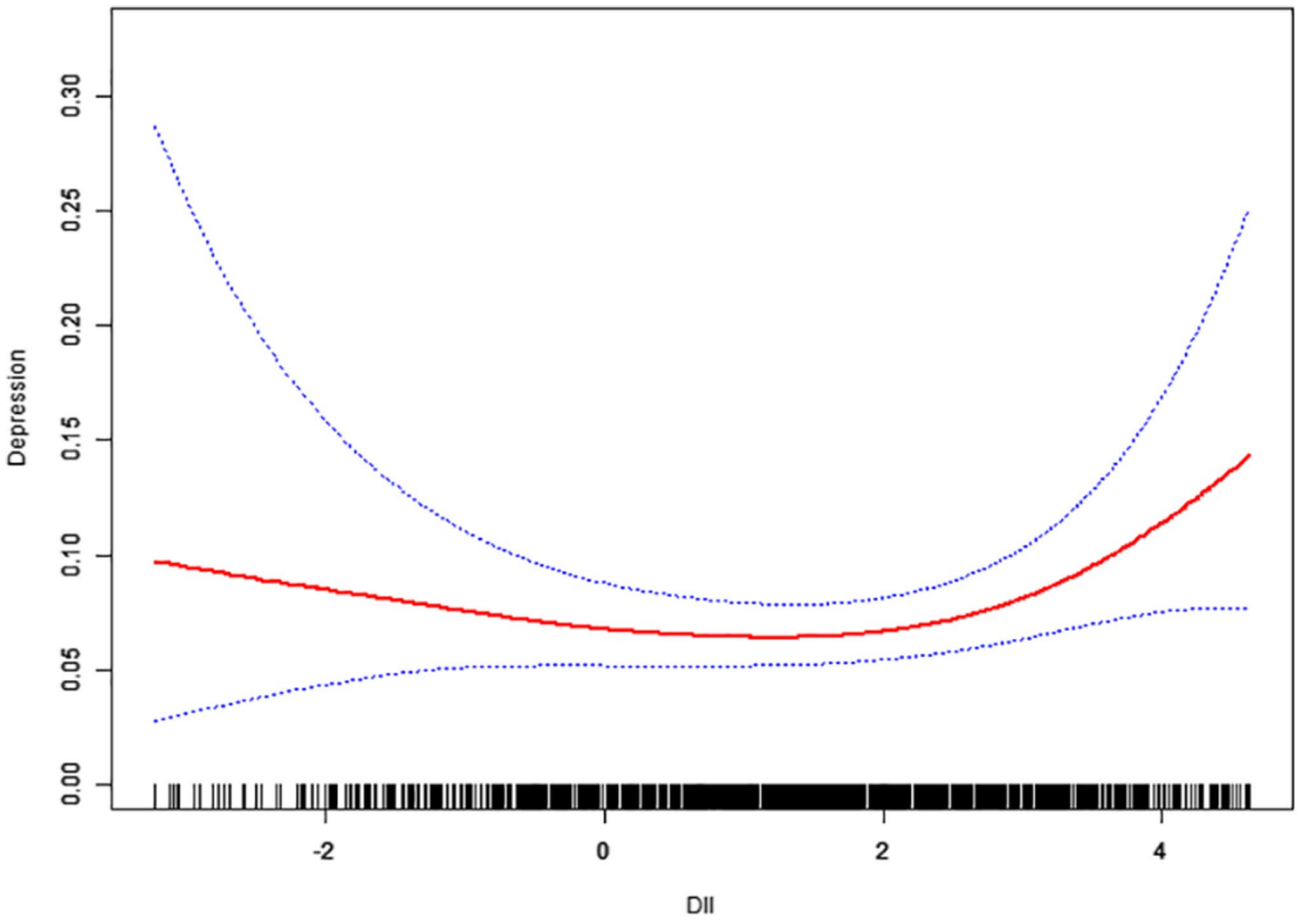
The association between Dietary Inflammatory Index and depression (PHQ-9 total score 10). Age, race, poverty income ratio, marital status, smoking status, education level, body mass index, white blood cell were adjusted.

We further observed a saturation effect between the DII and depression when we conducted smoothing curve fitting in the fully adjusted model (Figure 2).

Initial logistic regression (linear term for DII): In an adjusted weighted logistic model treating DII as a continuous linear variable, we found a positive but modest association between higher DII and odds of depression. The adjusted odds ratio was approximately 1.13 per 1.0 increase in DII (95% CI ~ 0.89–1.44), but this linear estimate alone did not reach statistical significance (*P* ≈ 0.31).

Non-linear relationship and threshold detection: Using a two-piecewise linear test, we observed that the log odds of depression were relatively flat or even slightly decreased from the lowest DII to a certain point and then began to increase more steeply beyond that point. This suggested a J-shaped curve. The likelihood ratio test for non-linearity was significant (*p* = 0.008 for non-linear spline terms), indicating that a straight-line model was insufficient (Table 3).

**Table 3:** Threshold effect analysis of the Dietary Inflammatory Index on depression using a two-piecewise linear regression model with NHANES sampling weights.

	PHQ-9 total score		Depression	
	β (95% CI)	*P*-value	OR (95% CI)	*P*-value
DII				
Fitting by standard linear model	−0.05 (−0.14, 0.24)	0.619	1.13 (0.89, 1.44)	0.310
Fitting by two-piecewise linear model				
Inflection point	2.87		2.87	
<2.87	−0.004 (−0.25, 0.24)	0.977	0.94 (0.69, 1.29)	0.715
>2.87	1.59 (0.02, 3.17)	0.047*	2.81 (1.07, 7.38)	0.039*
Log-likelihood ratio	0.018*		0.008*	

Age, race, poverty income ratio, marital status, body mass index, smoking status, and white blood cell count were adjusted. *p < 0.05.

β and 95% CI: from a weighted two-piecewise linear regression; CIs via weighted maximum likelihood (normal approximation).

OR and 95% CI: for binary outcomes, from a weighted logistic model; CIs by transforming ln(OR) with its SE and exponentiating.

To quantify this pattern, we implemented a piecewise (threshold) model with a change point at DII = 2.87 (the precise value identified by our algorithm maximizing model fit). The results from this two-piece logistic regression are as follows:

For DII values ≤2.87, the slope was essentially null to slightly negative. The adjusted OR for depression per 1-unit increase in DII in this lower range was approximately 0.94 (95% CI ~ 0.69–1.29; *p* = 0.715).

For DII values >2.87, a strikingly different picture emerged. The adjusted OR = 2.81 per 1-unit increase in DII was above 2.87 (95% CI 1.07–7.38; *p* = 0.039). This means that when the DII is already high (above ~2.9), an even more proinflammatory diet is associated with a substantially greater likelihood of depression. For example, a woman with a DII of +4.0 (very pro-inflammatory diet) had considerably higher predicted odds of depression than a woman with a DII of +3.0 (assuming both are above the threshold) after adjusting for other factors. To contextualize the magnitude, an increase of 1 DII unit in this high range more than doubled the odds of depression.

The log-likelihood ratio test results comparing the piecewise model to the simple linear model were highly significant (*p* = 0.008). This confirms that the two-piece model describes the data significantly better; i.e., the relationship between DII and depression is indeed non-linear (specifically, J-shaped).

### 4.3 DII and depressive symptom severity (linear regression results)

**Figure 3:**
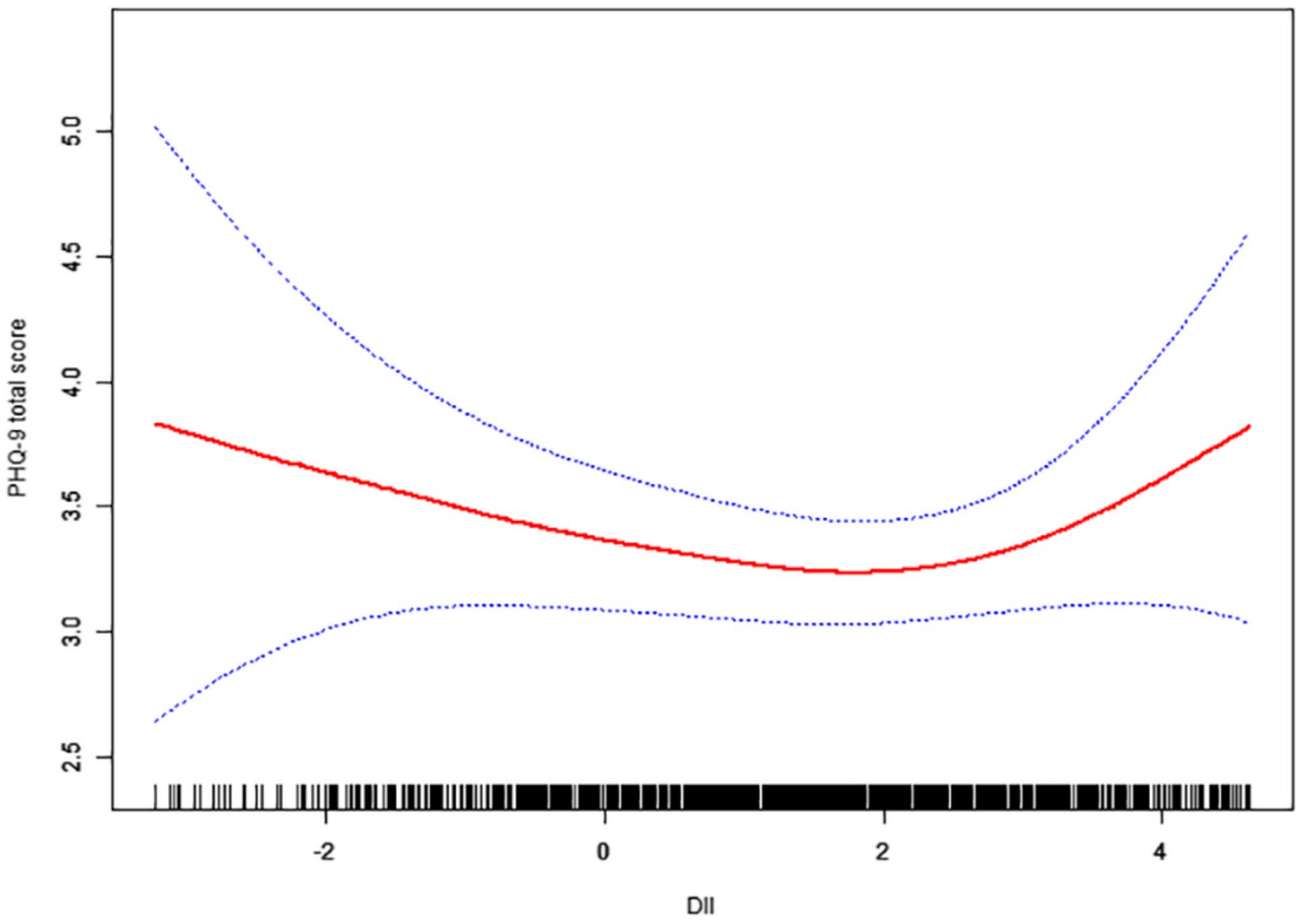
The association between Dietary Inflammatory Index and PHQ-9 total score. Age, race, poverty income ratio, marital status, smoking status, education level, body mass index, white blood cell were adjusted.

Using the PHQ-9 score as a continuous outcome yielded consistent findings (Figure 3).

In the adjusted weighted linear regression with a threshold, we found the following (Table 3):

DII ≤ 2.87: The association with the PHQ-9 score was small and not statistically significant. The adjusted *β* = −0.004 points on the PHQ-9 per 1-unit increase in DII in this range (95% CI –0.25 to +0.24; *p* = 0.977).

DII > 2.87: The adjusted *β* = +1.59 for PHQ-9 per 1-unit DII increase above the threshold (95% CI + 0.02 to +3.17, *p* = 0.047). This means that on average, within the high DII range, each additional point of the DII was associated with an increase of 0.83 in the PHQ-9 score.

### 4.4 Sensitivity analyses

In summary, across multiple analytic approaches, the key findings were consistent: only diets with high proinflammatory potential were linked to significantly elevated depression risk in pregnant/postpartum women, whereas diets with lower inflammatory potential did not show a clear relationship with depression in this group. This non-linear association was statistically supported and robust to various checks. These associations were further examined when we dichotomized age, and the results were consistent with the main findings (Supplementary Figures 1, 2).

We also validated the demographic and clinical characteristics of the participants in quartiles, and the results are shown in Supplementary Table 2. Through the quartile test, the direction of the core results was generally consistent with that of the tertiles. We triangulated the DII for the study design with the following main considerations in mind. First, it is possible to ensure a sufficient number of cases in each tertile to provide a more stable estimate while still preserving the exposure gradient. Second, the tertiles naturally mapped to ‘low’, ‘middle’ and ‘high’ proinflammatory diets, which is intuitive for clinicians. Third, reference was made to a study by Qin et al. (22) in this journal with the same parameters (*n* = 7,679), which also took a tertiles approach.

In addition, the PHQ-9 ranges from 0 to 27, with 0–4 indicating no depression and ≥5 suggesting depressive symptoms. Severity is further categorized as mild (5–9), moderate (10–14), or severe (15–27) (20); we also analyzed PHQ-9 ≥ 5 and PHQ-9 ≥ 15 as different cutoff points and found the same trend in the main results (Supplementary Table 3).

## 5 Discussion

In this cross-sectional study of pregnant and postpartum women in the U. S., we found that the inflammatory potential of the diet, as measured by the Dietary Inflammatory Index, is associated with the presence of depression in a non-linear, threshold-dependent manner. Women with highly proinflammatory diets had a significantly increased risk of depressive symptoms, whereas variations in dietary inflammation were not significantly linked to depression among those with anti-inflammatory diets. To our knowledge, this is one of the first studies focusing on the DII–depression link in a perinatal population, and our findings align with patterns observed in general adult samples while providing new insights into how diet might influence mental health during the sensitive pregnancy/postpartum period.

### 5.1 Comparison with previous studies

Our results corroborate and extend prior research on dietary inflammation and depression. Zhao et al. (16) examined a nationally representative sample of U. S. adults (encompassing all genders and a wide age range) and reported a J-shaped association between DII and depression, with a turning point at DII ≈ 2.74. Beyond that point, the odds of depression increased significantly (OR ~1.60 per unit increase in DII). We observed a very similar threshold (DII ~ 2.87) in our cohort of perinatal women, which is remarkable. This suggests that there may be a general level of dietary inflammatory load (around the high-2 range in DII score) beyond which the risk of depression increases in the human population. Interestingly, the magnitude of risk increase we found (OR ~2.81 per DII unit over threshold) was even greater than that in Zhao’s overall adult sample. There are a few possible explanations for this difference. First, pregnant and postpartum women may be particularly vulnerable to the mood effects of inflammatory stimuli due to the physiological and hormonal changes associated with pregnancy. The peripartum period involves complex immune modulation; for example, anti-inflammatory bias during mid-pregnancy shifts to a proinflammatory state during labor and early postpartum, which has been hypothesized to contribute to postpartum depression in some women. A highly proinflammatory diet could amplify this inherent inflammatory surge, potentially triggering or exacerbating depressive symptoms. Thus, our stronger association might reflect an interaction between diet and the unique biology of pregnancy. Second, it could be due to sample differences and study design—Zhao et al. (16) included all adults (mean age ~46 years, including men), whereas our sample comprised younger women; depression in young women might be more sensitive to diet, or our smaller sample might show a larger OR due to random variability. Nevertheless, the fact that both studies identified nearly the same critical DII range lends credibility to the threshold effect.

Our findings are also in agreement with a body of observational studies and meta-analyses linking proinflammatory diets to depression risk. A meta-analysis by Wang et al. (12) revealed a moderate overall association between a high DII and increased depression risk (pooled risk ratio of ~1.23 between the highest DII and lowest DII). Importantly, that meta-analysis revealed that the association was significant in women but not in men. Women might be more susceptible to the inflammatory effects of diet; this could be due to differences in the immune response or gut microbiota. Alternatively, it could be that their dietary patterns vary in ways that are specifically relevant to inflammation (23). In any case, our results reinforce that in women—and pregnant women, in particular—diet quality and inflammatory potential are relevant to mental health. Another cross-sectional analysis using NHANES data by Bergmans and Malecki (24) also revealed that higher DII was associated with greater odds of depression in adults, and there was no evidence of effect modification by sex in that study, although the sample was from the general population and did not explore non-linearity. Our work adds nuance by demonstrating the non-linear dose–response in a female reproductive-age subset.

In the context of pregnancy studies, a few related findings are worth highlighting. As mentioned, Yuan et al. (14) examined pregnant Chinese women and reported that those in the highest tertile of the DII had a significantly increased risk of prenatal depression (with an adjusted odds ratio of approximately 2.0 between the highest and lowest tertiles). This finding is consistent with our observation that very proinflammatory diets are linked to increased depression. Compared with these studies, our study first extended the study population to include parturients up to 18 months post-partum, which is more conducive to exploring the association between DII and depression throughout the perinatal period. We also found that there was a non-linear relationship and threshold effect between DII and depression during this perinatal period. These findings suggest that in the future, it may be necessary to focus on perinatal women whose Dietary Inflammatory Index exceeds a specific threshold and provide them with personalized dietary advice and nutritional services. Another study in pregnant women [Wang et al. (25), for example] revealed that healthier dietary patterns (akin to anti-inflammatory diets) were associated with lower depressive symptoms during pregnancy, although they did not specifically use DII. Our study contributes by leveraging a U. S. sample with a standardized measure (PHQ-9) and identifying a specific threshold, which could be practically useful. This suggests that clinicians might use the DII (which can be estimated via dietary recalls or questionnaires) to flag women above a certain score who could be at higher risk for depression and adverse outcomes for mothers and children (26, 27).

### 5.2 Biological plausibility

Proinflammatory diets can promote systemic inflammation, elevating cytokines like CRP, IL-6, and TNF-α. These molecules can disrupt serotonin production, impair neuroplasticity, and dysregulate the HPA axis, thereby fostering depressive symptoms. The perinatal period may represent a state of heightened vulnerability to these inflammatory insults. Our finding that only extremely proinflammatory diets (above a specific threshold) significantly increase depression risk suggests that the body’s regulatory mechanisms can buffer moderate inflammation, but become overwhelmed at high levels. This aligns with the slight, non-significant J-shape observed at the very low end of the DII, which may indicate a floor effect where non-dietary factors become primary drivers of risk once a diet is sufficiently anti-inflammatory, or where restrictive eating patterns counterbalance benefits. The key implication is that avoiding a highly proinflammatory diet is critical, whereas achieving a “perfect” anti-inflammatory diet may not be necessary for prevention.

### 5.3 Implications for intervention

If the associations are causal (an important caveat we discuss below), dietary modifications could help prevent or manage depression in pregnant and postpartum women (28). Encouraging anti-inflammatory foods and limiting proinflammatory components may reduce depression risk. Healthcare providers, such as obstetricians and dietitians, could assess diet using tools like the DII and counsel women with high DII scores on nutrition changes (e.g., increasing omega-3-rich foods, fruits, leafy greens, whole grains, and reducing processed foods). These dietary adjustments align with both mental health and general pregnancy nutrition guidelines. Anti-inflammatory diets, like the Mediterranean diet, have shown promise in improving depressive symptoms in general populations and should be tested in perinatal women (29, 30). This low-risk intervention could promote better mood outcomes and benefit both physical and mental health during pregnancy.

### 5.4 Public health significance

Perinatal depression is a major public health concern due to its high prevalence and the potential for negative outcomes, which can extend to the child (e.g., preterm birth, lower infant birth weight, impaired cognitive/emotional development in children of mothers with depression). Traditional risk factors for perinatal depression include personal or family psychiatric history, psychosocial stress, lack of social support, and hormonal changes. Our study adds a dietary pattern—specifically a proinflammatory diet—to the list of relevant factors. Importantly, diet is modifiable. Although one cannot change one’s genetic predisposition or easily alter one’s socioeconomic status, one can change what one eats with the right support and resources (15). This finding highlights a possibly actionable pathway for reducing depression burden. Community- and policy-level measures that improve diet quality among women of childbearing age (such as improving access to healthy foods, providing nutritional education, and integrating nutrition into maternal health programs) might have additional benefits for mental health outcomes. Further research to confirm causality could propel such initiatives.

### 5.5 Limitations

We must acknowledge several limitations of this study. First and foremost, the cross-sectional design limits our ability to infer causality or directionality. Exposure and outcomes were measured at the same time, and we cannot definitively establish that a proinflammatory diet preceded and contributed to the development of depression. Although our research hypothesis is based on the pathway of “diet influencing mood” (for example, through inflammation, oxidative stress or gut microbiota mechanisms), it is equally plausible that women who are depressed may change their eating habits because of their mood (for example, craving sugary “comfort foods” or having less motivation to prepare healthy meals), which could lead to a higher DII. The reverse path of “emotions influencing diet” also has a solid theoretical and empirical basis. Depressive states are often accompanied by changes in appetite, a craving for high-sugar and high-fat “comforting foods,” and a decrease in the motivation to prepare healthy meals. Such reverse causation is a concern (31). We attempted to mitigate this by adjusting for various confounders, but longitudinal data would be needed to disentangle the directionality. Some prospective studies in non-pregnant populations have shown that the baseline DII predicts future depressive symptoms, supporting a causal interpretation; however, we do not have that temporal evidence in our perinatal sample. Longitudinal studies or randomized trials are needed to establish a clearer cause–effect relationship.

Second, the measurement of diet via a single 24-h recall is a limitation. A one-day dietary snapshot may not capture an individual’s usual eating patterns. Day-to-day variability can be high. If a woman happens to have an unusual day of eating, her DII might be misclassified. Ideally, multiple days or a food frequency questionnaire over months would give a better estimate of long-term diet, as this is more relevant for influencing chronic inflammation. The use of NHANES data constrained us to available measures; some NHANES cycles did include a second 24-h recall on a subsample, but we did not incorporate that complexity here. The consequence of such measurement error would likely be an attenuation of true associations (bias toward the null) because random error in diet assessment dilutes correlations with outcomes. Therefore, our finding of a significant association at high DII scores suggests that the true effect could be even stronger if diet was measured perfectly. It is important to emphasize that the threshold was derived through statistical optimization based on the current dataset and that the data-driven results are exploratory in nature. The generalizability may be limited by a variety of factors, including the characteristics of the study population (such as age, region, and cultural background) and the assessment tools for depressive symptoms. Therefore, this threshold should not be considered directly as a clinical diagnostic or screening tool with universal applicability and may not be generalizable across populations or dietary assessments but rather as an initial reference value for future validation in different independent populations.

Another limitation of this study is the potential for residual or unmeasured confounders. Although we corrected for a range of sociodemographic and health factors, we lacked data on specific behaviors (e.g., omega-3 supplementation or vitamin D) and profound psychosocial determinants (e.g., trauma history, intimate partner violence, and social support). These unmeasured factors are an established risk factor for perinatal depression and may also be associated with dietary patterns, thus potentially confounding the observed associations. While we used education, income and marital status as imperfect indicators of socioeconomic circumstances, they do not directly reflect levels of stress or support. Future prospective studies specifically designed to investigate diet and mental health should prioritize direct and comprehensive assessments of these psychological and social domains to isolate the independent effects of diet.

We also acknowledge that the PHQ-9 measures current depressive symptoms, which may or may not meet clinical diagnostic criteria for major depressive disorder. Some women classified as “depressed” (PHQ-9 ≥ 10) might have transient symptoms or might be in treatment and improving. We did not verify the diagnoses via a psychiatric interview. However, the PHQ-9 is an accepted screening tool, and our threshold captures clinically meaningful cases; thus, we believe that it is a valid outcome measure for epidemiological purposes.

The RCS is a very powerful and flexible tool, but in the specific context of this study, the two-piecewise model was a better fit for the study hypotheses. Our study hypothesized that the association between DII and depression may not be linear and that there is a “threshold effect.” The two-piecewise model was able to directly and clearly estimate a specific threshold point. However, the RCS produces a smooth curve that, although perfectly demonstrating a non-linear relationship, does not directly provide a clear “threshold.” In addition, with a limited sample size, we believe that if a simple two-piecewise model can adequately describe the main feature of the data (i.e., a distinct inflection point), it may be more robust than a more complex model would be. Finally, the results from two-piecewise models are easy to present in tables (e.g., providing threshold points, effect sizes before and after the thresholds, and *p*-values). However, the RCS results are usually presented in graph form, as the shape of the plotted data may be sensitive to node position and number; thus, they are sometimes difficult to fully express in a table.

Despite these limitations, our study has notable strengths. It uses a relatively large sample of perinatal women with standardized data collection. We utilized a rigorous dietary index grounded in extensive research on the links between diet and inflammation. We accounted for many confounding variables and used advanced modeling to uncover a nuanced pattern in the data. The consistency of our findings with experimental knowledge of the role of inflammation in depression lends credibility to the results (16).

## 6 Conclusion

In conclusion, this study provides evidence that diet-related inflammation is associated with depression among pregnant and postpartum women. In particular, diets characterized by a high Dietary Inflammatory Index (indicating proinflammatory content) were linked to significantly greater odds of experiencing moderate-to-severe depressive symptoms. The relationship was non-linear, suggesting that consuming a highly proinflammatory diet may substantially increase depression risk, whereas consuming a moderately or strongly anti-inflammatory diet did not further lower the risk beyond a certain point. These findings highlight the importance of diet quality during pregnancy and the postpartum period, not only for physical health but also potentially for mental health.

## Data Availability

The original contributions presented in the study are included in the article/Supplementary material, further inquiries can be directed to the corresponding author.

## Footnotes

1
https://www.empowerstats.net/cn/
