# Supplementary material for: Associations between the Dietary Inflammatory Index and depression among pregnant and postpartum women: analysis of NHANES 2005–2018
Source: Front Nutr. 2025 Nov 27;12:1681491. doi: 10.3389/fnut.2025.1681491 (PMC12695599; doi:10.3389/fnut.2025.1681491)
Supplement: Supplementary file 1 [file Table_1.docx]

Supplementary Material

# **Supplementary TABLE 1** The food parameters used to calculate DII in this study.

.**Supplementary FIGURE 1** The association between DII and depression (PHQ-9 score ≥10) when dichotomized age.

**Supplementary FIGURE 2** The association between DII and PHQ-9 total score when dichotomized age.

**Supplementary TABLE 2** Demographic and clinical characteristics of the participants in quartiles.

**Supplementary TABLE 3** Threshold effect analysis of the Dietary Inflammatory Index on depression with different cut points.

# **Supplementary TABLE 1** The food parameters used to calculate DII in this study.

| **Food parameters** | **Raw inflammatory effect score** | **Overall inflammatory effect score** | **Global daily mean intake (units/d)** | **SD** |
| --- | --- | --- | --- | --- |
| Alcohol (g) | -0.278 | -0.278 | 13.98 | 3.72 |
| Beta-carotene (μg) | -0.584 | -0.584 | 3718 | 1720 |
| Caffeine (g) | -0.124 | -0.11 | 8.05 | 6.67 |
| Carbohydrates (g) | 0.109 | 0.097 | 272.2 | 40 |
| Cholesterol (mg) | 0.347 | 0.11 | 279.4 | 51.2 |
| Energy (kcal) | 0.18 | 0.18 | 2056 | 338 |
| Fe (mg) | 0.032 | 0.032 | 13.35 | 3.71 |
| Fiber (g) | -0.663 | -0.663 | 18.8 | 4.9 |
| Folic acid (μg) | -0.207 | -0.19 | 273 | 70.7 |
| Mg (mg) | -0.484 | -0.484 | 310.1 | 139.4 |
| MUFA (g) | -0.019 | -0.009 | 27 | 6.1 |
| n-3 fatty acids (g) | -0.436 | -0.436 | 1.06 | 1.06 |
| n-6 fatty acids (g) | -0.159 | -0.159 | 10.8 | 7.5 |
| Niacin (mg) | -1 | -0.246 | 25.9 | 11.77 |
| Protein (g) | 0.049 | 0.021 | 79.4 | 13.9 |
| PUFA (g) | -0.337 | -0.337 | 13.88 | 3.76 |
| Riboflavin (vitamin B2)  (mg) | -0.727 | -0.068 | 1.7 | 0.79 |
| Saturated fat (g) | 0.429 | 0.373 | 28.6 | 8 |
| Selenium (μg) | -0.191 | -0.191 | 67 | 25.1 |
| Thiamin (vitamin B1)  (mg) | -0.354 | -0.098 | 1.7 | 0.66 |
| Total fat (g) | 0.298 | 0.298 | 71.4 | 19.4 |
| Vitamin B12 (μg) | 0.205 | 0.106 | 5.15 | 2.7 |
| Vitamin B6 (mg) | -0.379 | -0.365 | 1.47 | 0.74 |
| Vitamin A (RE) | -0.401 | -0.401 | 983.9 | 518.6 |
| Vitamin C (mg) | -0.424 | -0.424 | 118.2 | 43.46 |
| Vitamin D (μg) | -0.446 | -0.446 | 6.26 | 2.21 |
| Vitamin E (mg) | -0.419 | -0.419 | 8.73 | 1.49 |
| Zinc (mg) | -0.313 | -0.313 | 9.84 | 2.19 |

Data was selected from https://www.cambridge.org/core/journals/public-health-nutrition/article/designing-and-developing-a-literaturederived-populationbased-dietary-inflammatory-index/30BE2C2295CE93DC6B54F9F9AD50CC68.

**Reference:** Shivappa N, Steck S E, Hurley T G, et al. Designing and developing a literature-derived, population-based dietary inflammatory index[J]. Public Health Nutr, 2014, 17(8): 1689-96.

**Supplementary FIGURE 1** The association between DII and depression (PHQ-9 score ≥10) when dichotomized age. Age, race, poverty income ratio, marital status, smoking status, BMI, and WBC were adjusted.


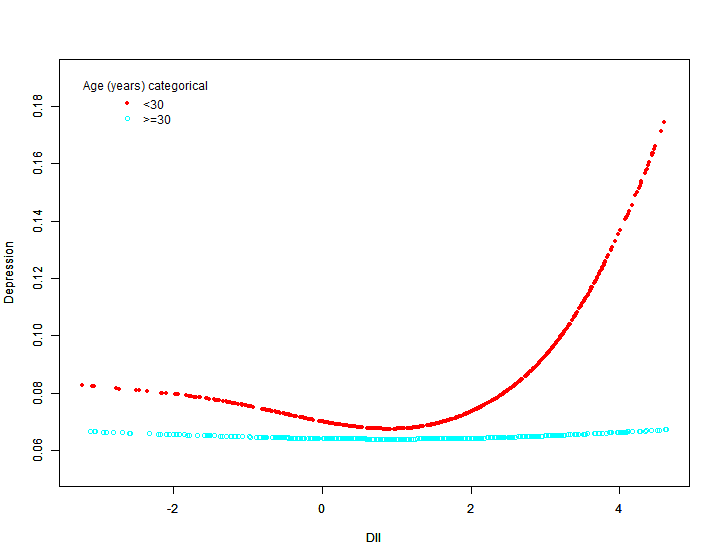


**Supplementary FIGURE 2** The association between DII and PHQ-9 total score when dichotomized age. Age, race, poverty income ratio; marital status, smoking status, BMI, and WBC were adjusted.


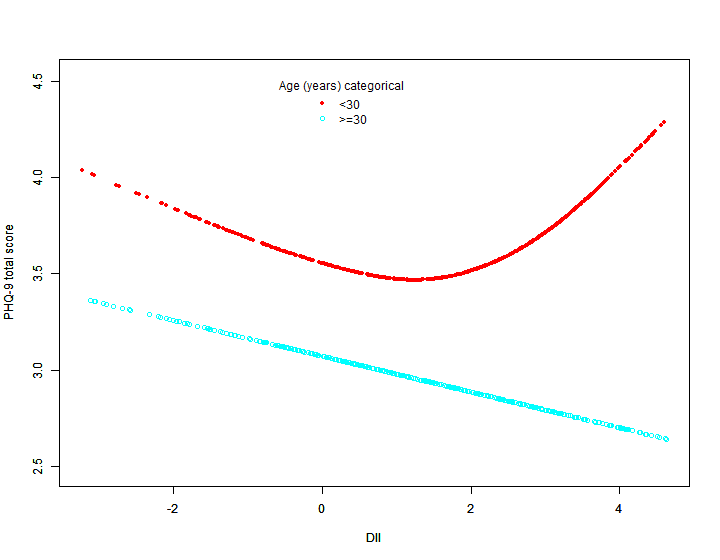


**Supplementary TABLE 2** Demographic and clinical characteristics of the participants in quartiles.

|  | **Q1**  **(−3.49−0.20)** | **Q2**  **(0.21−1.56)** | **Q3**  **(1.57−2.73)** | **Q4**  **(2.74−4.63)** | ***P* value** |
| --- | --- | --- | --- | --- | --- |
| Participants | 273 | 273 | 273 | 274 |  |
| Age (years) | 29.48 ± 5.91 | 28.70 ± 5.41 | 27.71 ± 5.56 | 27.55 ± 5.82 | <0.001 |
| BMI (kg/m^2^) | 28.44 ± 6.66 | 29.04 ± 6.41 | 29.76 ± 7.40 | 30.28 ± 7.41 | 0.013 |
| DII | -0.95 ± 0.86 | 0.91 ± 0.40 | 2.15 ± 0.33 | 3.47 ± 0.50 | <0.001 |
| WBC (1000 cells/µL) | 8.48 ± 2.56 | 8.61 ± 2.51 | 8.57 ± 2.53 | 8.16 ± 2.45 | 0.156 |
| PHQ-9 total score | 3.15 ± 3.59 | 3.24 ± 3.65 | 3.37 ± 3.80 | 3.65 ± 4.26 | 0.456 |
| Race/ethnicity (%) |  |  |  |  | 0.004 |
| Non-Hispanic White | 105 (38.46%) | 107 (39.19%) | 99 (36.26%) | 107 (39.05%) |  |
| Non-Hispanic Black | 37 (13.55%) | 33 (12.09%) | 58 (21.25%) | 63 (22.99%) |  |
| Mexican American | 72 (26.37%) | 71 (26.01%) | 61 (22.34%) | 58 (21.17%) |  |
| Other Hispanic | 22 (8.06%) | 23 (8.42%) | 29 (10.62%) | 29 (10.58%) |  |
| Other Race | 37 (13.55%) | 39 (14.29%) | 26 (9.52%) | 17 (6.20%) |  |
| Marital status (%) |  |  |  |  | 0.006 |
| Married/Living with partner | 231 (84.62%) | 222 (81.32%) | 211 (77.29%) | 195 (71.17%) |  |
| Widowed/Divorced/Separated | 9 (3.30%) | 14 (5.13%) | 13 (4.76%) | 14 (5.11%) |  |
| Never married | 33 (12.09%) | 37 (13.55%) | 49 (17.95%) | 65 (23.72%) |  |
| Poverty income ratio (%) |  |  |  |  | 0.002 |
| Poor | 57 (20.88%) | 73 (26.74%) | 82 (30.04%) | 106 (38.69%) |  |
| Nearly poor | 66 (24.18%) | 65 (23.81%) | 71 (26.01%) | 64 (23.36%) |  |
| Middle income | 63 (23.08%) | 62 (22.71%) | 61 (22.34%) | 51 (18.61%) |  |
| High income | 66 (24.18%) | 49 (17.95%) | 41 (15.02%) | 33 (12.04%) |  |
| Missing | 21 (7.69%) | 24 (8.79%) | 18 (6.59%) | 20 (7.30%) |  |
| Education level (%) |  |  |  |  | <0.001 |
| Below high school | 12 (4.40%) | 22 (8.06%) | 16 (5.86%) | 21 (7.66%) |  |
| High school | 81 (29.67%) | 91 (33.33%) | 126 (46.15%) | 122 (44.53%) |  |
| Above high school | 180 (65.93%) | 160 (58.61%) | 131 (47.99%) | 131 (47.81%) |  |
| Smoking status (%) |  |  |  |  | <0.001 |
| Never | 207 (75.82%) | 201 (73.63%) | 181 (66.30%) | 175 (63.87%) |  |
| Former | 44 (16.12%) | 38 (13.92%) | 41 (15.02%) | 29 (10.58%) |  |
| Current | 17 (6.23%) | 29 (10.62%) | 44 (16.12%) | 65 (23.72%) |  |
| Missing | 5 (1.83%) | 5 (1.83%) | 7 (2.56%) | 5 (1.82%) |  |
| Depression (%) |  |  |  |  | 0.098 |
| No | 256 (93.77%) | 256 (93.77%) | 255 (93.41%) | 244 (89.05%) |  |
| Yes | 17 (6.23%) | 17 (6.23%) | 18 (6.59%) | 30 (10.95%) |  |

BMI, body mass index; DII, Dietary Inflammatory Index; WBC, White Blood Cell.

Means (SD) for continuous variables. n(%) for categorical variables.

**Supplementary TABLE 3** Threshold effect analysis of the Dietary Inflammatory Index on depression with different cut points.

| DII | PHQ-9 total score | PHQ-9 ≥5 | PHQ-9 ≥10 | PHQ-9 ≥15 |
| --- | --- | --- | --- | --- |
|  | β (95% CI)  *P* value | OR (95% CI)  *P* value | OR (95% CI)  *P* value | OR (95% CI)  *P* value |
| Fitting by standard linear model | -0.02 (-0.16, 0.12)  0.7871 | 1.1 (0.92, 1.10)  0.8876 | 1.08 (0.93, 1.26)  0.3134 | 0.94 (0.72, 1.23)  0.6615 |
| Fitting by two-piecewise linear model |  |  |  |  |
| Inflection point | 2.87 | 2.87 | 2.87 | 2.87 |
| <2.87 | -0.14 (-0.31, 0.03)  0.1166 | 0.96 (0.86, 1.08)  0.5181 | 0.91 (0.75, 1.10)  0.3403 | 0.81 (0.58, 1.12)  0.2001 |
| >2.87 | 0.83 (0.11, 1.54)  0.0244 | 1.31 (0.86, 2.00)  0.2049 | 2.37 (1.32, 4.26)  0.0040 | 2.09 (0.72, 6.10)  0.1753 |
| Log-likelihood ratio | 0.018 | 0.208 | 0.008 | 0.152 |

Age, race, poverty income ratio, marital status, body mass index, smoking status, and white blood cell count were adjusted.
